# Teacher’s Autonomy Support and Engagement in Math: Multiple Mediating Roles of Self-efficacy, Intrinsic Value, and Boredom

**DOI:** 10.3389/fpsyg.2017.01006

**Published:** 2017-06-23

**Authors:** Jia Wang, Ru-De Liu, Yi Ding, Le Xu, Ying Liu, Rui Zhen

**Affiliations:** ^1^Beijing Key Laboratory of Applied Experimental Psychology, Faculty of Psychology, Beijing Normal UniversityBeijing, China; ^2^Graduate School of Education, Fordham University, New YorkNY, United States

**Keywords:** engagement, autonomy support, self-efficacy, intrinsic value, boredom, multiple mediating model

## Abstract

Previous studies have highlighted the impacts of environmental factors (teacher’s autonomy support) and individual factors (self-efficacy, intrinsic value, and boredom) on academic engagement. This study aimed to investigate these variables and examine the relations among them. Three structural equation models tested the multiple mediational roles of self-efficacy, intrinsic value, and boredom in the relation between teacher’s autonomy support and behavioral, emotional, and cognitive engagement, respectively, in math. A total of 637 Chinese middle school students (313 males, 324 females; mean age = 14.82) voluntarily participated in this study. Results revealed that self-efficacy, intrinsic value, and boredom played important and mediating roles between perceived teacher’s autonomy support and student engagement. Specifically, these three individual variables partly mediated the relations between perceived teacher’s autonomy support and behavioral and cognitive engagement, while fully mediating the relation between perceived teacher’s autonomy support and emotional engagement. These findings complement and extend the understanding of factors affecting students’ engagement in math.

## Introduction

Academic engagement has attracted the growing interest of educators and researchers, who have reported that it is a critical factor in ameliorating poor academic achievement, problem behaviors, and dropout (Klem and Connell, 2004; Archambault et al., 2009; Upadyaya and Salmela-Aro, 2013; Zhen et al., 2016). In the context of school, engagement usually consists of three aspects: behavioral, emotional, and cognitive, which describe how students behave, feel, and think during learning activities (Fredricks et al., 2004). The Math and Science Engagement Scales used to measure these three constructions in mathematics and science have been developed by Wang et al. (2016) and displayed adequate reliability and validity. Engagement is susceptible to environmental and individual factors (Fredricks et al., 2004). Given the important role of engagement in relation to achievement and life outcomes, it is essential to improve our understanding of the effect of these contextual and individual factors on fostering student engagement.

### Teacher’s Autonomy Support and Engagement

Among the numerous environmental factors, teacher’s behaviors play an important role in enhancing or diminishing student engagement (Skinner et al., 2008). Recently, many researchers have focused on teacher’s autonomy support and have highlighted its positive effects on student engagement (e. g., Reeve et al., 2004; Skinner et al., 2008; Chen et al., 2015; Hospel and Galand, 2016; Jang et al., 2016; Yu et al., 2016). Teachers provide autonomy support to their students by providing them with choices, fostering their understanding and interest about the learning subjects, and encouraging them to think independently and critically (Assor et al., 2002). Grounded in this definition, the scale measuring autonomy-affecting teacher behaviors was developed by Assor et al. (2002) and it had satisfactory reliability and validity. According to self-determination theory (SDT, Ryan and Deci, 2000; Deci and Ryan, 2008), teacher’s autonomy support, as the source of students’ basic psychological need support, is critical for student engagement.

### Boredom and Engagement

Another key factor influencing engagement is achievement emotion. The control-value theory of achievement emotions (Pekrun, 2006) has proposed that achievement emotions are important for students’ cognitive, motivational, and regulatory processes in their learning. Recent researchers have reported that achievement emotions are significantly correlated to different types of engagement, such as behavioral effort, use of learning strategies, self-regulation of learning, and emotional engagement (Isen, 2000; Pekrun et al., 2002; Wolters, 2003; Luo et al., 2014; Tze et al., 2014). Achievement emotions include both activity emotions that tie directly to learning activities (e. g., enjoyment and frustration) and outcome emotions that tie directly to the outcomes of these activities (e. g., hope and anxiety; Pekrun, 2006). It is of particular importance to explore specific emotions, since each may have different antecedents and consequences. Among the specific emotions, boredom has been shown to be one of the most commonly experienced emotions in school contexts (Tze et al., 2014). A survey showed that middle school students experienced boredom during about one third of their class time (Larson and Richards, 1991). Boredom in achievement settings has been defined as a kind of negative and passive emotion, which was related to unpleasant experience and reduction of physiological activation (Pekrun et al., 2010). Based on this definition, an 11-item scale measuring boredom was developed by Dong and Yu (2007), which showed satisfactory psychometric properties among Chinese adolescents. As a commonly experienced negative emotion, the negative impact of boredom warrants exploration. However, researchers have focused far less attention on boredom than on other achievement emotions (Pekrun et al., 2010; Tze et al., 2016). For example, more than 1000 researchers have investigated the causes and effects of academic anxiety, but less than 40 studies have discussed boredom in school settings (Pekrun et al., 2002).

Recently, boredom has received increasing but still sporadic attention from researchers, and some studies have shown that boredom has a negative impact on learning outcomes (Pekrun et al., 2010, 2011; Ahmed et al., 2013; Tulis and Fulmer, 2013). Pekrun et al. (2010, 2011) conducted a series of cross-sectional and longitudinal studies regarding boredom in school contexts. Results indicated that boredom was positively associated with other negative achievement emotions (e. g., anxiety) and negatively associated with positive achievement emotions (e. g., enjoyment) as well as achievement outcomes, such as intrinsic motivation, effort, self-regulatory learning, and academic performance (Pekrun et al., 2010, 2011). More recently, Ahmed et al. (2013) found a negative association between boredom and the use of effective learning strategies in math. Tulis and Fulmer (2013) found that boredom was detrimental for persistent engagement in math. These findings suggested that boredom would have negative impact on academic engagement.

### Linking Teacher’s Autonomy Support and Boredom: Effects of Self-efficacy and Intrinsic Value

In the control-value framework, teacher’s autonomy support is one of the most important social antecedents of boredom (Pekrun, 2006, 2007). Some empirical studies provided evidence for this argument. For instance, Tze et al. (2014) investigated the developmental trend of boredom during a course. They found that perceived teacher’s autonomy support negatively predicted students’ class-related boredom experiences (Tze et al., 2014). Furthermore, Leptokaridou et al. (2014) examined the effects of teacher’s autonomy support on student motivations for participating in school physical education and found that autonomy-supportive teaching effectively reduced students’ experiences of boredom.

According to the control-value theory of achievement emotions, the impact of teacher’s autonomy on boredom should be mediated by the cognitive appraisals of subjective control and subjective values (Pekrun, 2006). Subjective control refers to expectations and attributions about the causal effects of the self on actions and outcomes, for example, expecting that current efforts will lead to positive academic performance. Subjective values contain intrinsic value, which refers to students’ beliefs about the importance and interest of the learning activity *per se*, and extrinsic value, which refers to students’ perceptions of the instrumental usefulness of an action or outcomes of attaining other goals. As argued by the control-value theory, different achievement emotions are determined by different types of appraisal antecedents. With regard to boredom, as an activity-related emotion, appraisals of action control should play a more important role than outcome control. In other words, students experience boredom when they perceive a sense of lack control over the activities of learning, not a sense of lack of control over the outcomes. Self-efficacy, which refers to one’s perception of his/her capacity to perform a learning task, was the most popular term used to represent action control (Pekrun, 2006). Empirical studies have measured self-efficacy, using the Self-Efficacy Scale of the Motivated Strategies for Learning Questionnaire (MSLQ, Pintrich and De Groot, 1990), as an indicator of subjective control and found that self-efficacy was significantly correlated with boredom (Pekrun et al., 2011). Within subjective value, a lack of intrinsic value of learning activities is more critical than a lack of extrinsic value for stimulating boredom (Pekrun et al., 2010). A student will experience boredom when he/she perceives that learning activities are uninteresting and have little relevance to personal identity (Pekrun et al., 2010). Pekrun et al. (2011) investigated the relation between intrinsic value, measured by the intrinsic value scale of MSLQ (Pintrich and De Groot, 1990) and boredom. The result showed that intrinsic value and boredom were negatively correlated (Pekrun et al., 2011). In short, self-efficacy and intrinsic value should be two core predictors of academic boredom.

With regard to the relation between teacher’s autonomy support and the two cognitive appraisals (self-efficacy and intrinsic value), the SDT proposed that teacher’s autonomy support can provide fulfillment of student’s basic psychological needs for autonomy, competence and relatedness, and consequently enhance their perceived competence and intrinsic motivation (Deci et al., 1981). Recently, the effects of teacher’s autonomy support on student’s self-efficacy and intrinsic value have been empirically examined. Using a 1-year longitudinal design, Jungert and Koestner (2015) found that both teachers’ and parents’ autonomy support positively predicted self-efficacy among high school students. Similarly, Ng et al. (2015) found positive associations between perceived teacher’s autonomy support and self-efficacy. Moreover, they investigated the relation between the motivation beliefs and autonomy support and found that perceived teacher’s autonomy support was a predictor of intrinsic value. All these findings indicated that teacher’s autonomy support could enhance self-efficacy and intrinsic value. In short, we could postulate that autonomy support could positively influence the experiences of boredom by enhancing the two cognitive appraisals of self-efficacy and intrinsic value.

### Multiple Mediation Models

Both teacher’s autonomy support and boredom have an impact on academic engagement; moreover, autonomy support can alleviate the experiences of boredom through increased self-efficacy and intrinsic value. Thus, there should be a multiple mediating mechanism between teacher’s autonomy support and engagement. Specifically, autonomy support might influence self-efficacy and intrinsic value, therefore influence boredom, and then influence engagement.

Notably, the impacts of self-efficacy and intrinsic value on engagement include not only indirect effects by triggering boredom, but also direct effects (Chouinard et al., 2007). On the one hand, students with high self-efficacy tend to expend greater effort on their learning activities and persist longer in the face of challenges and setbacks (Bandura, 1977). High self-efficacy contributes to high academic engagement (Linnenbrink and Pintrich, 2003; Patrick et al., 2007; Sakiz et al., 2012; Martin and Rimm-Kaufman, 2015). On the other hand, high intrinsic value will lead students to use more cognitive strategies and to manage their efforts more effectively (Pintrich and De Groot, 1990). González et al. (2016) found that intrinsic value positively predicted use of self-regulatory and deep processing strategies and persistence in an undergraduate statistics class. Zhen et al. (2016) found that intrinsic value had a direct positive impact on all three dimensions of academic engagement (behavioral, emotional, and cognitive engagement). Thus, self-efficacy and intrinsic value could both be considered as proximal predictors of students’ engagement and distal predictors that influence the experiences of boredom.

Taken together, the impact of teacher’s autonomy support, self-efficacy, intrinsic value, and boredom on academic engagement are complex and inter-correlated. On the one hand, all of these factors have direct effects on engagement. On the other hand, the influence of autonomy, as an important environmental factor, on engagement is mediated by the individual factors of self-efficacy, intrinsic value, and boredom. Hence, the main aim of this study was to investigate the multiple mediating effects of self-efficacy, intrinsic value, and boredom on the relation of perceived teacher’s autonomy support and academic engagement.

## The Present Study

As discussed above, teacher’s autonomy support, self-efficacy, intrinsic value, academic boredom, and their relations with student engagement have been examined respectively. However, to date, the concurrent and systematic effects of these factors on engagement have not been explored. In addition, there are few empirical studies focusing on the mediating effects among these variables. For example, the control-value theory suggests that the impact of environmental factors on achievement emotions should be mediated by control and value appraisals (Pekrun, 2006). However, the mediating effects of self-efficacy and intrinsic value between teacher’s autonomy support and boredom have not been investigated by empirical studies. In addition, the multiple mediating roles of self-efficacy, intrinsic value, and boredom on the relation between teacher’s autonomy support and student engagement has not been explored by empirical studies as well.

This study analyzed a model of structural relations between teacher’s autonomy support, self-efficacy, intrinsic value, academic boredom, and engagement in the math domain among Chinese middle school students. Mathematics is a basic discipline and a critical school subject across the world. Particularly in China, mathematics education receives widespread attention (Zhen et al., 2016). In this context, it is of great importance to investigate Chinese students’ engagement in mathematics.

**FIGURE 1:**
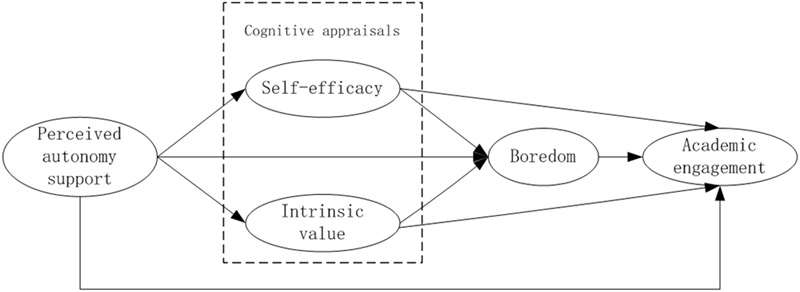
The overall hypothesized model of relations among study variables.

Based on the SDT (Ryan and Deci, 2000; Deci and Ryan, 2008), the control-value theory (Pekrun, 2006), and the previous findings outlined above, the present study proposed a multiple mediation model that postulated that teacher’s autonomy support would predict self-efficacy and intrinsic value, which, in turn, would predict the experiences of boredom that subsequently predict academic engagement. Specifically, as shown in **Figure 1**, the hypotheses were: (a) Perceived autonomy support, self-efficacy, and intrinsic value would positively predict engagement, while boredom would negatively predict engagement; (b) autonomy support, self-efficacy, and intrinsic value would negatively predict boredom; (c) the influence of autonomy support on boredom would be mediated by the two cognitive appraisals (self-efficacy and intrinsic value); (d) the influence of self-efficacy and intrinsic value on engagement would be mediated by boredom; (e) self-efficacy, intrinsic value, and boredom would mediate the positive influence of autonomy support on engagement.

Notably, engagement is a multidimensional concept, which includes behavior, emotion, and cognition (Fredricks et al., 2004). Behavioral engagement refers to involvement in academic and class-related activities; emotional engagement is conceptualized as positive and negative emotional reactions to teachers, peers, and learning activities; cognitive engagement is defined as the willingness to exert effort to comprehend complex knowledge and to master skills (Fredricks et al., 2004; Wang et al., 2016). Considering the three types of engagement represent distinct and unique constructs that may be affected by the above factors to different degrees (Wang et al., 2016), the present study conducted three structural equation models (SEMs) to respectively examine the influences of the above factors on behavioral, emotional, and cognitive engagement.

## Materials and Methods

### Participants and Procedure

A total of 637 Chinese middle school students (324 females and 313 males) in Hubei province voluntarily participated in this study. The students were on average 14.82 years of age (*SD* = 0.96) with a range from 11 to 17 years old. This study was approved by the Research Ethics Committee of Beijing Normal University. All subjects gave written informed consent in accordance with the Declaration of Helsinki. The investigation was conducted during regularly scheduled classes. Participants were informed about the purpose of this study and the voluntary nature of participation, and then completed a series of questionnaires within 45 min. The questionnaires included the measures of perceived teacher’s autonomy support, self-efficacy, intrinsic value, boredom, and engagement in math.

### Measures

#### Perceived Teacher’s Autonomy Support

Perceived teacher’s autonomy support was measured using 13 items adapted from the scales measuring autonomy-affecting teacher behaviors (Assor et al., 2002). These items measured students’ perceived teacher’s autonomy-supportive behaviors, including providing choice, fostering understanding and interest, allowing criticism, and encouraging independent thinking. We made some modifications in wording to specifically target the domain of mathematics (e.g., “When I am doing something that interests me, my math teacher gives me enough time to finish it,” “My teacher explains why it is important to study mathematics,” “My math teacher listens to my opinions and ideas.”). Participants responded on a Likert-type scale ranging from 1 (*not true at all*) to 5 (*very true*). The internal reliability of the scale in this study was adequate (α = 0.87).

#### Self-efficacy

Self-efficacy was measured using a nine-item scale adapted from the MSLQ (Pintrich and De Groot, 1990). All nine items were revised to fit the mathematical context in this study (e.g., “I expect to do very well in math class,” “I know that I will be able to learn the materials for math class”). Participants responded on a 5-point Likert-type scale (1 = *not true at all of me* to 5 = *very true of me*). The Cronbach alpha for this scale was 0.87.

#### Intrinsic Value

The nine-item intrinsic value scale from the MSLQ was used to measure students’ intrinsic value in mathematics (Pintrich and De Groot, 1990). The present study revised this scale to fit the mathematical context (e.g., “I think what we are learning in math class is interesting,” “It is important for me to learn what is being taught in math class”). Each item was rated using a 5-point Likert-type scale (1 = *not true at all of me* to 5 = *very true of me*). The scale in this study had satisfactory internal consistency (α = 0.86).

#### Academic Boredom

Academic boredom was measured by 11 items adapted from the Academic Emotions Questionnaire (Dong and Yu, 2007). The questionnaire was designed for a Chinese context and was inspired by previous work, including the School Failure Tolerance Scale (SFT; Clifford, 1988), the Multidimensional School Anger Inventory (MSAI; Smith et al., 1998), and the Achievement Emotions Questionnaire (AEQ; Pekrun et al., 2002). This questionnaire was suitable for measuring Chinese students’ boredom experiences. Sample items of this questionnaire were “I’m not interested in mathematics,” “I feel sleepy when learning mathematics,” and “I think mathematics is boring.” Participants responded on a 5-point Likert-type scale (1 = *not true at all of me* to 5 = *very true of me*). Cronbach’s alpha was 0.93 for this scale.

#### Engagement

Engagement in mathematics was assessed by using three subscales of the Math and Science Engagement Scales (Wang et al., 2016). Participants responded on a Likert-type scale ranging from 1 (*not true at all*) to 5 (*very true*). Behavioral engagement was measured by eight items, such as “I put effort into learning math” (α = 0.76, the current study); emotional engagement was measured by 10 items, such as “I enjoy learning new things about math” (α = 0.86, the current study); and cognitive engagement was measured by eight items, such as “I think about different ways to solve a problem” (α = 0.72, the current study).

### Data Analysis

Descriptive statistics and correlation analysis of the study variables were conducted using SPSS 17.0. There were no missing data. We considered behavioral engagement, emotional engagement, and cognitive engagement as dependent variables and conducted three two-step processes (Anderson and Gerbing, 1988) using Mplus 7.0 to test the hypothesized models. Specifically, we first tested the measurement model of the constructs, and then we tested the structural model to explore the relation of the variables. The model fit was evaluated by multiple indicators (Hu and Bentler, 1999; Tabachnick and Fidell, 2006; Byrne, 2010): the indicator χ*^2^*/*df*, the Comparative Fit Index (CFI), the Tucker Lewis Index (TLI), the root mean squared error of approximation (RMSEA), and the standardized root mean squared residual (SRMR). The model can be considered as a good fit when the χ^2^/*df* is below 2, the CFI and the TLI are closed to 0.95, the RMSEA is closed to 0.06, and the SRMR is closed to 0.08. The model can be considered as acceptable when the χ^2^/*df* is below 5, the CFI and the TLI are over 0.90, and the RMSEA and the SRMR are below 0.10 (Bentler and Bonett, 1980; Hu and Bentler, 1999; Byrne, 2010; Tabachnick and Fidell, 2006). Furthermore, a bootstrap estimation procedure with 5,000 bootstrap samples was conducted to examine the mediating hypotheses (Preacher and Hayes, 2008).

## Results

### Preliminary Analysis

The results of descriptive analysis as well as the correlations between the study variables are shown in **Table 1**. Each of the three types of engagement in math was positively correlated to perceived teacher’s autonomy support, self-efficacy, and intrinsic value, as well as negatively correlated to academic boredom. Furthermore, academic boredom was negatively correlated to perceived teacher’s autonomy support, self-efficacy, and intrinsic value. Moreover, the perceived teacher’s autonomy support, self-efficacy, and intrinsic value were positively correlated with each other.

**Table 1:** Descriptive statistics and correlations for major variables.

	Statistic		Correlations					
Variable	*M*	*SD*	1	2	3	4	5	6
1. Autonomy support	3.13	0.72	–					
2. Self-efficacy	2.93	0.81	0.30^∗∗^	–				
3. Intrinsic value	3.30	0.65	0.24^∗∗^	0.33^∗∗^	–			
4. Boredom	2.37	0.91	-0.23^∗∗^	–0.45^∗∗^	-0.36^∗∗^	–		
5. Behavioral engagement	3.51	0.68	0.29^∗∗^	0.47^∗∗^	0.40^∗∗^	0.60^∗∗^	–	
6. Emotional engagement	3.32	0.80	0.30^∗∗^	0.56^∗∗^	0.38^∗∗^	0.76^∗∗^	0.71^∗∗^	–
7. Cognitive engagement	3.18	0.68	0.27^∗∗^	0.44^∗∗^	0.34^∗∗^	0.57^∗∗^	0.66^∗∗^	0.63^∗∗^

*^∗∗^*p* < 0.01.*

### Measurement and Structural Model

Each of the measurement models in this study consisted of six latent factors (perceived teacher’s autonomy support, self-efficacy, intrinsic value, boredom, and one type of engagement) as well as 40, 42, and 40 indicators, respectively. Specifically, the measured variables were three indicators (three dimensions): providing choice (PC: five items), fostering understanding and interest (FU&I: four items), allowing criticism and encouraging independent thinking (AC&EIT: four items), respectively, on autonomy support; nine items on self-efficacy and intrinsic value; eleven items on boredom; eight items on behavioral engagement; and cognitive engagement; and ten items on emotional engagement.

**FIGURE 2:**
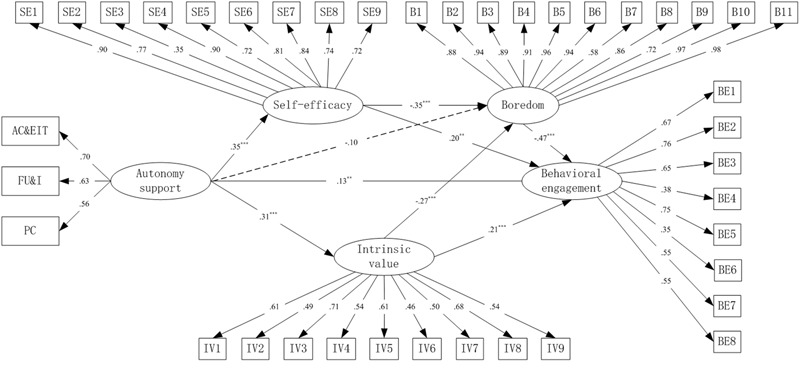
Full mediation model for behavioral engagement as dependent variable. PC, providing choice; FU&I, fostering understanding and interest; AC&EIT, allowing criticism and encouraging independent thinking; SEl–SE9, nine items of self-efficacy; IV1–IV9, nine items of intrinsic value; B1–B11, 11 items of boredom; BE1–BE8, eight items of behavioral engagement. ^∗∗∗^*p* < 0.001; ^∗∗^*p* < 0.01.

**FIGURE 3:**
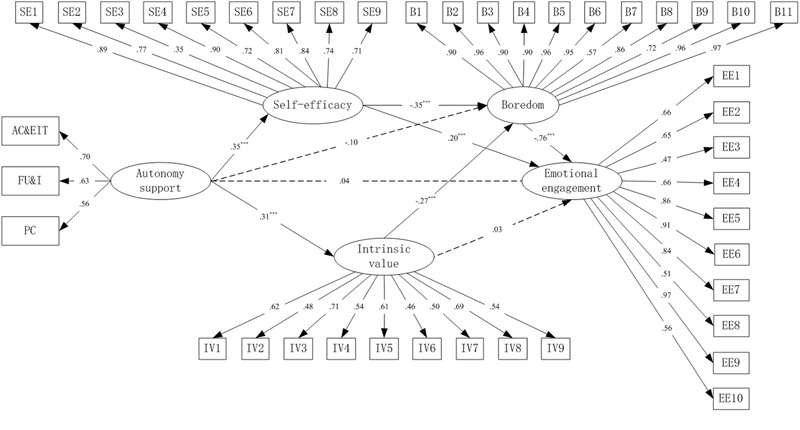
Full mediation model for emotional engagement as dependent variable. PC, providing choice; FU&I, fostering understanding and interest; AC&E IT, allowing criticism and encouraging independent thinking; SE1–SE9, nine items of self-efficacy; IV1–IV9, nine items of intrinsic value; B1–B11, 11 items of boredom; EE1–EE10, 10 items of emotional engagement. ^∗∗∗^*p* < 0.001.

**FIGURE 4:**
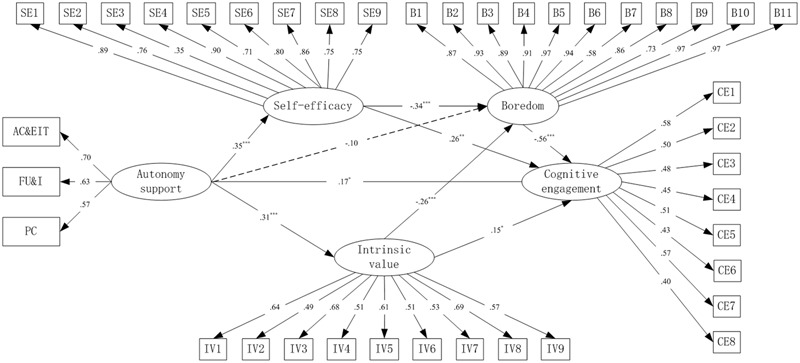
Full mediation model for cognitive engagement as dependent variable. PC, providing choice; FU&I, fostering understanding and interest; AC&EIT, allowing criticism and encouraging independent thinking; SE1–SE9, nine items of self-efficacy; IV1–IV9, nine items of intrinsic value; B1–B11, 11 items of boredom; CE1–CE8, eight items of cognitive engagement.^∗∗∗^*p* < 0.001; *^∗∗^p <* 0.01; ^∗^*p* < 0.05.

Overall, the results of confirmatory factor analysis (CFA) showed acceptable fit for the structural model with behavioral engagement as the dependent variable: χ*^2^* = 1271.993, *df* = 710, *p* < 0.01, χ*^2^*/*df* = 1.792, CFI = 0.949, TLI = 0.944, RMSEA = 0.035, SRMR = 0.047; for the structural model with emotional engagement as dependent variable: χ*^2^* = 1519.197, *df* = 776, *p* < 0.01, χ*^2^*/*df* = 1.958, CFI = 0.943, TLI = 0.937, RMSEA = 0.039, SRMR = 0.053; and for the structural model with cognitive engagement as dependent variable: χ*^2^* = 1207.592, *df* = 689, *p* < 0.01, χ*^2^*/*df* = 1.753, CFI = 0.953, TLI = 0.947, RMSEA = 0.034, SRMR = 0.047, respectively. The factor loadings were all statistically significant (*p* < 0.001, shown in **Figures 2–4**) and achieved the factor-loading criterion of 0.35 (Byrne, 2010), which indicated that the latent variables were adequately measured by these indicators.

Considering the acceptable fit of the measurement models, three SEM analyses were conducted to test the hypothesized models. Results showed that all of the three hypothesized models adequately fit the data. Fit indexes for the model with behavioral engagement as dependent variable were: χ*^2^* = 1271.993, *df* = 710, *p* < 0.01, χ*^2^*/*df* = 1.792, CFI = 0.949, TLI = 0.944, RMSEA = 0.035, SRMR = 0.047; for the model with emotional engagement as dependent variable were: χ*^2^* = 1519.197, *df* = 776, *p* < 0.01, χ*^2^*/*df* = 1.958, CFI = 0.943, TLI = 0.937, RMSEA = 0.039, SRMR = 0.053; and for the model with cognitive engagement as dependent variable were: χ*^2^* = 1207.592, *df* = 689, *p* < 0.01, χ*^2^*/*df* = 1.753, CFI = 0.953, TLI = 0.947, RMSEA = 0.034, SRMR = 0.047, respectively.

The overall structural models with standardized regression weights are portrayed in **Figures 2–4**, respectively. The direct path coefficients from perceived teacher’s autonomy support to behavioral (β = 0.13, *p* < 0.01) and cognitive engagement (β = 0.17, *p* < 0.05) were significant. However, the direct path coefficient from perceived teacher’s autonomy support to emotional engagement was non-significant (β = 0.04, *p* > 0.05). Furthermore, both self-efficacy and intrinsic value positively predicted behavioral (self-efficacy: β = 0.20, *p* < 0.01; intrinsic value: β = 0.21, *p* < 0.001) and cognitive engagement (self-efficacy: β = 0.26, *p* < 0.01; intrinsic value: β = 0.15, *p* < 0.01). However, for emotional engagement, only self-efficacy (β = 0.20, *p* < 0.001) but not intrinsic value (β = 0.03, *p* > 0.05) was a significant direct predictor. Boredom negatively predicted behavioral (β = -0.47, *p* < 0.001), emotional (β = -0.76, *p* < 0.001) and cognitive (β = -0.56, *p* < 0.001) engagement, respectively. In addition, self-efficacy (β = -0.35, -0.35, and -0.34, respectively, *p* < 0.001) and intrinsic value (β = -0.27 in all of the three models, *p* < 0.001) negatively predicted boredom in all of the three models, whereas the direct path from perceived teacher’s autonomy support to boredom was non-significant (β = -0.10 in all of the three models, *p* > 0.05). The predictors explained moderate to high proportions of variance in behavioral (*R^2^* = 0.61), emotional (*R^2^* = 0.82), and cognitive (*R^2^* = 0.77) engagement, respectively.

### Test for Mediation

Finally, the mediating relations of the study variables were tested using a bootstrapping method (*n* = 5,000 bootstrap samples). The significance of the indirect effects was determined at the level of 0.05 in this study; the indirect effect was considered statistically meaningful if the estimates of the 95% confidence interval did not contain zero. As shown in **Table 2**, the indirect effects in all of the three models were statistically significant, which supported our hypotheses concerning the mediating mechanisms among the study variables. Specifically, the relation between perceived teacher’s autonomy support and boredom was fully mediated by the two cognitive appraisals (self-efficacy: β = -0.12; intrinsic value: β = -0.08). Furthermore, boredom partially mediated the influences of self-efficacy and intrinsic value on behavioral (self-efficacy: β = 0.16; intrinsic value: β = 0.13) and cognitive engagement (self-efficacy: β = 0.19; intrinsic value: β = 0.15). For emotional engagement, boredom partially mediated the effect of self-efficacy (β = 0.26), while fully mediated the effect of intrinsic value (β = 0.21). More important, the individual factors (self-efficacy, intrinsic value, and boredom) partially mediated the relation between perceived teacher’s autonomy support and behavioral (β = 0.10) and cognitive engagement (β = 0.11), while fully mediated the relation between autonomy support and emotional engagement (β = 0.16).

**Table 2:** Bootstrap analyses of the significance of mediation.

Model	Standardized		95% CI mean indirect	
pathways	indirect effect	*SE*	effect (lower and upper)	
**Model with behavioral engagement as dependent variable**				
AS-SE-B	-0.120^∗∗∗^	0.023	-0.166,	-0.075
AS-IV-B	-0.084^∗∗∗^	0.024	-0.131,	-0.037
SE-B-BE	0.162^∗∗∗^	0.028	0.108,	0.216
IV-B-BE	0.125^∗∗∗^	0.029	0.069,	0.181
AS-SE-B-BE	0.056^∗∗∗^	0.012	0.032,	0.080
AS-IV-B-BE	0.039^∗∗^	0.012	0.016,	0.063
**Model with emotional engagement as dependent variable**				
AS-SE-B	-0.120^∗∗∗^	0.023	-0.164,	-0.085
AS-IV-B	-0.085^∗∗∗^	0.024	-0.132,	-0.038
SE-B-EE	0.264^∗∗∗^	0.036	0.193,	0.335
IV-B-EE	0.207^∗∗∗^	0.042	0.125,	0.289
AS-SE-B-EE	0.091^∗∗∗^	0.017	0.057,	0.126
AS-IV-B-EE	0.065^∗∗^	0.019	0.028,	0.102
**Model with cognitive engagement as dependent variable**				
AS-SE-B	-0.120^∗∗∗^	0.023	-0.165,	-0.075
AS-IV-B	-0.082^∗∗∗^	0.023	-0.127,	-0.036
SE-B-CE	0.192^∗∗∗^	0.038	0.117,	0.266
IV-B-CE	0.148^∗∗∗^	0.036	0.078,	0.219
AS-SE-B-CE	0.067^∗∗∗^	0.015	0.037,	0.097
AS-IV-B-CE	0.045^∗∗^	0.014	0.017,	0.074

*AS, perceived teacher’s autonomy support; SE, self-efficacy; IV, intrinsic value; B, boredom; BE, behavioral engagement; EE, emotional engagement; CE, cognitive engagement.*

*^∗∗^*p* < 0.01, ^∗∗∗^*p* < 0.001.*

## Discussion

The present study aimed at exploring the systematic relations between academic engagement and its environmental and individual predictors. SEM analysis was used to simultaneously estimate the direct or indirect relationships among study variables. The results supported most of the hypotheses in this study.

### Direct Relations

Perceived teacher’s autonomy support positively predicted students’ behavior and cognitive engagement. These findings concurred with the SDT (Ryan and Deci, 2000; Deci and Ryan, 2008) and previous studies on the relations of autonomy support and academic engagement (Reeve et al., 2004; Skinner et al., 2008; Chen et al., 2015; Hospel and Galand, 2016; Jang et al., 2016; Yu et al., 2016). Self-efficacy and intrinsic value enhanced the behavioral and cognitive types of academic engagement, which further supported previous conclusions highlighting the positive role of these two individual factors in engagement (Linnenbrink and Pintrich, 2003; Patrick et al., 2007; Sakiz et al., 2012; Martin and Rimm-Kaufman, 2015; González et al., 2016; Zhen et al., 2016). Students who experienced more boredom reported less behavioral and cognitive engagement in math. These findings supported the notion that boredom hinders engagement (Pekrun et al., 2010; Pekrun et al., 2011; Ahmed et al., 2013; Tze et al., 2014, 2016). With regard to emotional engagement, only self-efficacy and boredom had direct effects: Students with high self-efficacy and low level of boredom reported more emotional engagement in math.

The control-value theory argued that cognitive appraisals of subjective control and value were antecedents of achievement emotions (Pekrun, 2006). The present study found that two specific indices of subjective control and value (self-efficacy and intrinsic value) negatively predicted boredom, which was consistent with the control-value theory. In addition, although previous studies found that autonomy support was negatively related to boredom (Leptokaridou et al., 2014; Tze et al., 2014), the results of this study showed that a direct relation between autonomy support and boredom was non-significant when controlling for self-efficacy and intrinsic value.

### Mediated Relations

As expected, and in line with the control-value theory (Pekrun, 2006), this study found that the influence of teacher’s autonomy support on boredom was fully mediated by the cognitive appraisals of self-efficacy and intrinsic value: Middle school students who perceived that their math teachers supported their autonomy experienced low levels of boredom in math because they felt they had the capacity for learning mathematics and believed mathematics was important and interesting.

With regard to the relations between self-efficacy and engagement, self-efficacy positively predicted all three types of engagement, and these associations were partly mediated through boredom: Students with high self-efficacy engaged more in math (in part) because they felt low levels of boredom. Similar to self-efficacy, the effects of intrinsic value on behavioral and cognitive engagement were partly mediated by boredom. However, the effect of intrinsic value on emotional engagement was fully mediated by boredom: Students who believed that mathematics was important and interesting displayed positive emotional reactions to their learning activities mostly because they experienced less boredom.

Previous studies have found positive associations between teacher’s autonomy support and student engagement (Reeve et al., 2004; Skinner et al., 2008; Chen et al., 2015; Hospel and Galand, 2016; Jang et al., 2016; Yu et al., 2016), while in this study the intense and positive relations between autonomy support and engagement were mostly mediated by individual factors including self-efficacy, intrinsic value, and boredom. The results indicated that teacher’s autonomy-supportive behaviors both directly and indirectly enhanced students’ behavioral involvement and cognitive effort in math through influencing individual factors. For emotional engagement, the indirect effect of autonomy support was significant whereas the direct effect was not, indicating full mediation. In other words, teacher’s autonomy-supportive behaviors *per se* cannot directly influence students’ emotional reactions to learning. It appeared that teacher’s autonomy support made students feel more confident, value math more, and experience less boredom in math, thus students reported higher emotional engagement in math.

### Implications

Previous studies found that both environmental factors, such as teacher’s autonomy support, and individual factors, such as self-efficacy, intrinsic value, and boredom, had impact on engagement. The present study extended previous work by examining the concurrent and systematic effects of the above variables on engagement. The path, perceived teacher’s autonomy support → self-efficacy and intrinsic value → boredom → engagement, illuminated the multiple mediating roles of cognitive appraisals and boredom in the relations between autonomy support and engagement. The results in this study indicated that teacher’s autonomy support had different effects on different types of engagement. The influences of autonomy support on behavioral and cognitive engagement were statistically significant and partially mediated by self-efficacy, intrinsic value, and boredom, whereas the relation between autonomy support and emotional engagement was fully mediated by the above factors. These results further supported the viewpoint that behavioral, emotional, and cognitive engagement are unique and different constructs (Wang et al., 2016). In addition, the findings could serve as practical guidelines for teachers to organize an effective learning environment that fosters student engagement in math. Specifically, teachers can increase autonomy support of their students by providing them with more choices, advocating the value of learning math, and encouraging criticism. In this way, students will have more self-efficacy and intrinsic value, subsequently experiencing less boredom and engaging more in math.

### Limitations and Future Study

This study assessed three main components of teacher’s autonomy-supportive behavior (providing choice, fostering relevance, and encouraging independent thinking) and used these three components as indicators of the latent construct autonomy support. Future work could explore the individual effects of each autonomy-supportive component on engagement to further understand the relation between specific autonomy-supportive behavior and engagement. Furthermore, other relevant constructs should be considered in future studies, such as “teacher’s autonomy-suppressing behaviors” (Assor et al., 2002) and “classroom structure” (Hospel and Galand, 2016). This study assessed students’ perceived autonomy support. Future study could measure teacher-reported autonomy support (e. g., Soenens et al., 2012) or experimentally manipulate teacher’s autonomy-supportive behavior (e.g., Leptokaridou et al., 2014).

This study measured students’ boredom in math. Previous studies have found that there were two types of academic boredom: learning-related boredom and class-related boredom (Pekrun et al., 2011). Future work could investigate the differences in the relations between each type of boredom and engagement, such as the work of Tze et al. (2014). In addition, this study assessed three main types of engagement (behavioral, emotional, and cognitive), which are most often included in previous research (e. g., Skinner et al., 2008; Hospel and Galand, 2016; Zhen et al., 2016). According to Wang et al. (2016), social engagement that refers to social interactions with peers and adults in the learning context is an important type of engagement as well. Therefore, it is necessary for future studies to extend the understanding of how environmental and individual factors influence students’ social engagement in math.

## Author Contributions

All the coauthors are participants in the data collection and analysis, writing and revising the manuscript.

## Conflict of Interest Statement

The authors declare that the research was conducted in the absence of any commercial or financial relationships that could be construed as a potential conflict of interest. The reviewer DK and handling Editor declared their shared affiliation, and the handling Editor states that the process nevertheless met the standards of a fair and objective review.
